# To Our Exchanges

**Published:** 1875-01

**Authors:** 


*To our Exchanges.*

We owe many thanks to our cotemporaries for their courtesy and kindness during the past year, and hope we may continue to merit their fraternal good-will. We have received all of our exchanges for January, for which we tender to each one our warmest thanks, and promise to send in return, punctually every month, a copy of the SOUTHERN MEDICAL RECORD. We should be more united in our efforts to elevate the standard of medical education, and to advance the science of medicine, and the true development and application of its important aims and results.

Medical ethics should not be regarded as a code of laws for the jurisdiction of the fraternity of any one section or State, but should be maintained for the protection of every member of the brotherhood, from shore to shore, who is true and loyal to its principles.

With cordial fraternal greetings of the New Year, we trust another annual cycle will bring to light many hidden truths in the art of healing that will be of incalculable benefit to mankind, and elevate to a still higher altitude the standard of true medical theory and practice. P.

